# Seroprevalence of transfusion transmissible viral infections (HIV, HBV and HCV) among voluntary blood donors at University of Gondar Comprehensive Specialized Hospital, Gondar; Northwest Ethiopia

**DOI:** 10.1186/s12879-019-3950-2

**Published:** 2019-05-08

**Authors:** Abiye Tigabu, Tigist Engda, Feleke Mekonnen

**Affiliations:** 1 Department of Medical Microbiology, School of Biomedical and Laboratory Sciences, College of Medicine and Health Sciences, University of Gondar, P O.box: 196, Gondar, Ethiopia; 2 Department of Medical Laboratory Sciences, School of Health Sciences, College of Medicine and Health Sciences, Bahir Dar University, Bahir Dar, Ethiopia

**Keywords:** Seroprevalence, Transfusion-transmissible viral infections, HIV, HBV, HCV, Blood donors

## Abstract

**Background:**

Human immunodeficiency virus, hepatitis B virus and hepatitis C virus are among the greatest threats to blood safety for the recipient. They are also the leading cause of death, chronic and life-threatening abnormalities. Therefore, this study was aimed to assess the Sero-prevalence of HIV, Hepatitis B and C virus among blood donors at the University of Gondar Comprehensive Specialized Hospital.

**Methods:**

A retrospective cross-sectional study was used to estimate the seroprevalence of HIV, Hepatitis B and C virus among blood donors at the University of Gondar Comprehensive Specialized Hospital from May–July 2018. Screening of HIV, HBV, and HCV was done by using the Enzyme-Linked ImmunoSorbent Assay. Records of 5983 first time blood donors were collected and reviewed by using a checklist from registration book. Data was entered in statistical package EP Info version 3.5.1, and data cleaned and analyzed using the statistical package SPSS version 16.0.

**Results:**

Of 5983 blood donors, 85.5% (5118/5983) donors were males and 14.5% (865/5983) were females. The median age was 27 years and the highest blood donations age category was between 20 to 51.2% (29/5983) followed by 30 to 39 years of age, 21.6% (1295/5983). The prevalence of HIV, HBV and HCV infections were 2.5% (95% CI: 1.07–2.398), 4.1% (95% CI: 0.461–1.053) and 1.6% (95% CI: 0.845–3.354), respectively. HIV infection was significantly associated with gender (*p* = 0.021, x^2 =^ 5.358) and HCV infection with age group (*p* = 0.003, x^2^ = 17.673). Of all donated blood, 8.2% (489/5983) had serological evidence for at least one of the screened pathogens and 58 (0.96%) of them had multiple infections.

**Conclusions:**

This study showed a significant prevalence of HIV, HBV, and HCV among blood donors, 2.5% (147/5983), 4.1% (244/5983) and 1.6% (98/5983), respectively. Therefore, strict selection of blood donors with an emphasis on getting voluntary blood donors, and highly sensitive and specific tests for screening of blood donors for HIV, HBV, and HCV using standard methods are highly recommended to ensure the safety of blood for the recipient.

## Background

Human immunodeficiency virus (HIV), hepatitis B virus (HBV) and hepatitis C virus (HCV) are the greatest threats to blood safety because of their prolonged presence in the blood as a carrier or latent state. They are the leading cause of death, chronic and life-threatening abnormality. Blood transfusion accounts for 5–10% of HIV infections in sub-Saharan Africa [1]. The frequency of HIV infection among blood donors varies significantly between different countries of the world. There is a strong association between infection with the human immunodeficiency virus and hepatitis C virus infection [2]. HIV infection occurs worldwide and is endemic in central Africa. Transmission occurs through sexual contact, exposure to contaminated blood or blood products, and perennially. Human immunodeficiency virus is one member of the human retrovirus and is associated with progressive immune deficiency accompanied by a wide range of opportunistic infections [3].

Hepatitis B virus (HBV) is generally recognized as highly infectious and associated with long term occurrence of disease and death due to complications like cirrhosis, portal hypertension, and Hepato-cellular carcinoma. Approximately greater than 2 billion people have been infected by HBV and 350 million individual people have chronic infection [1, 4]. The common high-risk groups for HBV infections are parenteral drug abusers, institutionalized persons, health care personnel, multiply transfused patients, organ transplant patients, hemodialysis patients, highly promiscuous persons, sexual transmission and newborn infants born to mothers with hepatitis B virus [5]. The virus is highly infectious and relatively easy to be transmitted from one infected person to another by blood to blood contact, during birth, unprotected sex, and by sharing needles and has a relatively increase the prevalence in the tropics [6].

Hepatitis C virus (HCV) was identified in 1989 as the major agent of post-transfusion hepatitis previously designated under the name of “non-A, non-B hepatitis [7]. It is a blood-borne pathogen transmitted most efficiently by percutaneous membrane exposure to contagious blood. It is estimated that 60% of hepatitis C cases are related to injection drug use (IDU) [8]. HCV infection is highly recognized as a major healthcare problem throughout the world and around 130 to 170 million people are chronic carriers of this virus in the world, with an average seroprevalence estimated at 2.2%. This rate varies from country to country: it is very low in Europe, higher in Southeast Asia and Africa [7]. HCV causes 85% persistent infection, of this 70% of them develops chronic hepatitis, resulting in cirrhosis of the liver within 20 years and hepatocellular carcinoma (HCC) in a further 10 years [9]. Hepatitis C virus is associated with the seropositivity of the human immunodeficiency virus infection [2]. There are some reports which indicate the burden of transfusion-transmissible viral infections in Ethiopia. However, there are several localities including the current study area in which prevalence and trends yet not determined. Therefore, this study was aimed to determine the seroprevalence of HIV, Hepatitis B and C virus among blood donors at the University of Gondar Comprehensive Specialized Hospital., Gondar, Ethiopia.

## Methods

### Study design, study area, data collection

A retrospective cross-sectional study was conducted to determine the seroprevalence of HIV, Hepatitis B and C virus among first time voluntary blood donors at the University of Gondar compressive specialized Hospital from May–July 2018. Records of 5983 blood donors were collected and reviewed by using a checklist from registration book at blood banking unit of the hospital. Information concerning HIV, HBV and HCV test results, age, sex and occupation of blood donors recorded from September 2014–September 2017 in the registration book was collected using a data collection format.

### Laboratory detection methods

Screening for HIV, HBV and HCV was done by using the Enzyme-Linked ImmunoSorbent Assay (ELISA) (HIV1/2: Vironostika HIV Uni-Form II Ag/Ab fourth generation ELISA, Bio-Merieux, Boxtel, Netherlands; HBsAg: a third generation ELISA, Hepanostika HBsAg UNi-Form II, Bio-Merieux, Boxtel, Netherlands; HCV: Human anti-HCV third generation ELISA, HumanGasellschaft for Biochemical and diagnostic MbH, Germany). Test protocol and result interpretation were done according to the manufacturer instruction.

### Data management and statistical analysis

Quantitative data was entered in statistical package EP-Info version 3.5.1, and data cleaned and analyzed using the statistical package SPSS version 16.0. Frequency distribution, percentages and summary statistics were used to describe the study population in relation to relevant variables. Odds Ratio (OR) was computed to assess statistical association, and the significance of statistical association was assured using *p*-value < 0.05 at 95% confidence interval (CI).

## Results

### Socio-demographic characteristics of blood donors

A total of 5983 consecutive first time blood donors were screened at University of Gondar Teaching Hospital blood bank unit during the study period. Of these, 5118 (85.5%) donors were males and 865 (14.5%) were females. The median age of these blood donors was 27 years (range 15–68 years) and the highest blood donations age category was between 20 to 29 (51.2%), followed by 30 to 39 years of age (21.6%) (Table 1).

**Table 1:** Frequency of blood donors by age groups and gender at University of Gondar Compressive Specialized Hospital, North West Ethiopia, 2018

Characteristics	Frequency (No)	Percent (%)
Age group (years)		
< 20	509	8.5
20–29	3063	51.2
30–39	1295	21.6
40–49	740	12.4
50–59	341	5.7
60 & above	35	0.6
Total	5983	100.0
Gender		
Male	5118	85.5
Female	865	14.5
Total	5983	100.0

### Seroprevalence of HIV, HBV, and HCV

The overall prevalence rates of HIV, HBV, and HCV were 2.5% (147/5983), 4.1% (244/5983) and 1.6% (98/5983), respectively. Of all donated blood during the study period, 8.2% (489/5983) had serological evidence for at least one of the screened pathogens, and 0.97% (58/5983) of them was multiple infections. Of these, 88.2% (431/489) and 11.8% (58/489) had single and multiple infections, respectively. Among those with multiple infections, the most common combination was HIV-HBV 50% (29/58), then followed by HBV-HCV 25.9% (15/58), HIV-HCV 24.1% (14/58) and HIV-HBV-HCV 8.6% (5/58). The seroprevalence of HIV in male and female donors is 1.94% (116/5983) and 0.52% (31/5983), respectively. The difference is statistically significant (*p* = 0.021). HIV prevalence rate among age category < 20, 20 to 29, 30 to 39, 40 to 49, 50 to 59 and 60 & above was 0.2% (12/5983), 1.1% (66/5983), 0.74% (44/5983), 0.25% (15/5983), 0.15% (9/5983) and 0.02% (1/5983), respectively. Age with different category does not have a statistically significant association with HIV (*p* = 2.51) (Table 2).

**Table 2:** Seroprevalence of HIV among different age and gender categories at University of Gondar Compressive Specialized Hospital in Northwest Ethiopia, 2018

Variable	HIV status		Chi-square	Crude OR	CI	*P*-value
	Negative (%)	Positive (%)				
Gender						
Male	5002 (83.6%)	116 (1.94%)	5.264	1.6	1.07–2.398	0.022
Female	834 (13.9%)	31 (0.52%)	_	_	_	_
Age category						
< 20	497 (8.3%)	12 (0.2%)	6.504	_	_	0.260
20–29	2997 (50%)	66 (1.1%)	0.84	0.912	0.489–1.700	0.772
30–39	1251 (21%)	44 (0.74%)	1.300	1.457	0.763–2.781	0.254
40–49	725 (12.1%)	15 (0.25%)	0.155	0.857	0.398–1.846	0.693
50–59	332 (5.51%)	9 (0.15%)	0.067	1.123	0.468–2.694	0.795
60 & above	34 (0.57%)	1 (0.02%)	0.035	1.218	0.154–9.647	0.852

**Fig. 1:**
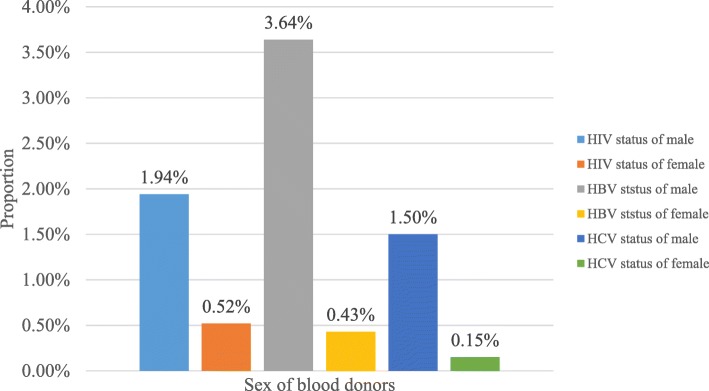
Comparison of HIV, HBV and HCV among Gender at University of Gondar Compressive Specialized Hospital, Northwest Ethiopia, 2018

The seroprevalence of HBV was higher among males, 3.64% (218/5983) compared to females, 0.43% (26/5983) but the difference is not statistically significant (*p* = 0.085). Its prevalence among age category < 20, 20 to 29, 30 to 39, 40 to 49, 50 to 59 and 60 & above was 0.32% (19/5983), 1.9% (113/5983), 0.9% (54/5983), 0.7% (41/5983), 0.27% (16/5983) and 0.02% (1/5983), respectively. Age with different category do not have statistically significant association with HBV (*p* = 0.319) (Table 3). Similarly, the seroprevalence of HCV in male was 1.5% (89/5983) compared to females 0.15% (9/5983). The difference is not statistically significant (*p* = 0.134). Its seroprevalence of HCV with age group, < 20, 20 to 29, 30 to 39, 40 to 49, 50 to 59 and 60 & above was 0.03% (2/5983), 0.7% (42/5983), 0.55% (33/5983), 0.25% (15/5983), 0.07% (4/5983) and 0.03% (2/5983), respectively. The difference is statistically significant (*p* = 0.003) (Table 4). HBV is the most important infection especially in male, 3.64% to compare with female, 0.43%. And HIV is contributing higher percentage in male, 1.94% to compare with female, 0.52%. Similarly, HCV is slightly higher in male, 1.5% to compare with female, 0.15% but it was lower than the total infection of HIV and HVB infection (Fig. 1).

**Table 3:** Seroprevalence of HBV among different Age and Gender categories at University of Gondar Compressive Specialized Hospital, Northwest Ethiopia. 2018

Variable	HBV status		Chi-square	Crude OR	CI	*P*-value
	Negative (%)	Positive (%)				
Gender						
Male	4900 (82%)	218 (3.64%)	2.942	0.697	0.461–1.053	0.086
Female	839 (14%)	26 (0.43%)	_	_	_	_
Age group						
< 20	490 (8.2%)	19 (0.32%)	5.810	_	_	0.325
20–29	2950 (49.3%)	113 (1.9%)	0.70	1.318	0.171–10.146	0.791
30–39	1241 (20.7%)	54 (0.9%)	0.067	1.302	0.177–9.599	0.795
40–49	699 (11.7%)	41 (0.7%)	0.146	1.479	0.199–11.010	0.702
50–59	325 (5.4%)	16 (0.27%)	0.452	1.994	0.266–14.934	0.502
60 & above	34 (0.57%)	1 (0.02%)	0.242	1.674	0.215–13.014	0.623

**Table 4:** Seroprevalence of HCV among different age and gender categories at University of Gondar Compressive Specialized Hospital, Northwest Ethiopia, 2018

Variable	HCV status		Chi-square	Crude OR	CI	*P*-value
	Negative (%)	Positive (%)				
Gender						
Male	5029 (84.1%)	89 (1.5%)	2.192	1.697	0.845–3.354	0.139
Female	856 (14.3%)	9 (0.15%)	_	_	_	_
Age category						
< 20	507 (8.5%)	2 (0.03%)	15.579	_	_	0.008
20–29	3021 (50.1%)	42 (0.7%)	3.016	3.524	0.850–14.604	0.082
30–39	1262 (21.1%)	33 (0.55%)	6.711	6.629	1.585–27.727	0.010
40–49	725 (12.1%)	15 (0.25%)	4.818	5.245	1.194–23.035	0.028
50–59	337 (5.6%)	4 (0.07%)	1.607	3.009	0.548–16.519	0.205
60 & above	33 (0.55%)	2 (0.03%)	7.230	15.346	2.094–112.54	0.007

### Trends of HIV, HBV and HCV Seroprevalence

**Fig. 2:**
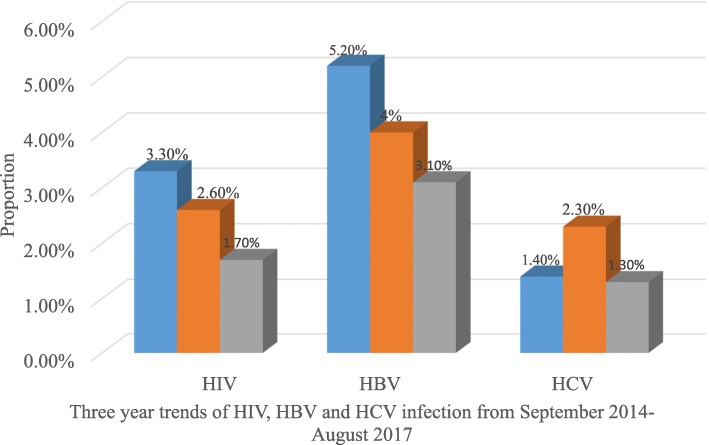
Trends of HIV, HBV and HCV infection by years at University of Gondar Compressive Specialized Hospital, Northwest Ethiopia, 2018

Over the three years study periods, the seroprevalence of HIV was decreased from 3.3% in September 2014–August 2015 to 2.6% in September 2015–August 2016 and 1.7% in September 2016–August 2017. Similarly, the prevalence of HBV decreased progressively from 5.2% in September 2014–August 2015 to 4.0% in September 2015 –August 2016 and 3.1% in September 2016 – August 2017. However, the seroprevalence of HCV was increased from 1.4% in September 2014–August 2015 to 2.3% in September 2015 – August 2016 but slightly decreased 1.3% in September 2016 – August 2017 (Fig. 2).

## Discussion

One of the main recommendations of the WHO to achieve a safe and sufficient blood supply is the collection of blood from voluntary regular non-remunerated donors who have a lower risk of TTIs compared to family replacement and commercial donors. In this study, the overall prevalence of HIV among blood donors in the last three years at University of Gondar compressive specialized Hospital blood bank, 2.5% is lower than previous studies conducted Ethiopia, 3.8 and 11.79% [16, 25], in Cameroon, 2.9 and 4.1% [12, 23], in Dares salaam, 3.8% [13], in Mozambique, 8.5% [24], in Nigeria, 2.8, 6.2 and 3.1% [17, 26, 27]. However, the overall prevalence of HIV in this study, 2.5% was higher as compared to the prevalence of HIV in Ethiopia, 1.4, 0.25 and 0.1% [18, 37, 39], in Netherland, 0.06% [10], in Nepal, 0.12% [11], in the University Clinics of Kinshasa of Democratic Republic of the Congo, 2.2% [2], in Saudi Arabia, 0% [14], in Istanbul, Turkey, 0.008% [28], in Kathmandu, Nepal, 0.007% [29], in Pakistan, 0.007, 0.25, 0.017 and 0.09% [30, 35, 38, 40], in Delhi, 0.56% [31], in Kashan, Iran, 0% [32], in southeastern Anatolia, 0.0004% [33], in India, 0.27 and 0.39% [34, 42], in Koudougou, 2.21% [36], in Lahore, 0.05% [41]. This variation of seroprevalence of TTIs from other studies might be due to due to difference in the characteristics of the study population, geographical distribution and diagnostic techniques.

Higher HIV prevalence rates were observed in the age groups of 20–29 (1.1%) followed by 30–39 (0.74%). The prevalence of HIV detected in the age groups of 20–29 was higher as compared to other age groups. Hence, the risk of HIV infection in age groups 20–29 is 1.5 times higher when compared with age groups less than 20 years. The higher prevalence of HIV in the age group 20–29 might be due to peoples in this age group have high-risk behaviors such as multiple sex partners, intravenous drug abuse and unprotected sexual intercourse. The seroprevalence of HIV was statistically significant among male donors, 1.94% (116/5983) compared to female, 0.52% (31/5983) (*P* = 0.021). The higher in HIV positive male donors might be due to their increased vulnerability to HIV infection as result of biological, economic and social disadvantages.

The prevalence of HBV in this study among University of Gondar Hospital blood bank blood donors, 4.1% in line with the previous study in Pakistan, 4.0% [30]. The prevalence of HBV among blood donors in the present study, 4.1% was higher when compared with studies on blood donors in Netherlands, 0.215% [10], and in Nepal, 0.46 and 0.007% [11, 29], in Saudi Arabia, 1.5% [14], in Istanbul, Turkey, 1.76% [28], in Delhi, 2.23% [31], in Kashan, Iran, 0.5% [32], in southeastern Anatolia, 3.17% [33], in India, 1.38 and 1.41% [34, 42], in Pakistan, 2.51, 2.35 and 1.9% [35, 38, 40], in Ethiopia, 1.2% [37], in Lahore, 1.7% [41], and lower than a study in Ethiopia, 25 and 10.9% [25, 39], in Cameroon, 10.3 and 10.1% [12, 23], in Dares salaam, 8.8% [13], in Mozambique, 10.6% [24], in Nigeria, 10 and 18.6% [26, 27], in Koudougou, 14.96% [36]. The reasons for the relatively lower or higher rate of seroprevalence of HBV in this study as compared to other studies might be the improvement in diagnostic technology might make current screening reagents to be more specific and reliable; the economic status of the country and the geographical differences in prevalence.

The seroprevalence of HBV detected in the age groups of 20–29, 1.9% (113/5983) and 30–39 years, 0.9% (54/5983) was higher than other age groups. The risk of HBV infection among age groups 20–29 was 1.5 times higher compared with other age groups. This study was similar to the previous reports in Kathmandu, Nepal [11], Amhara and Tigray regional states, Ethiopia [15]. HBV seroprevalence was highest in these age groups might be due to the different risk behaviors in these age groups. HBV infection by gender distribution showed that the prevalence of HBV among males, 3.64% was higher than the females, 0.43%. A higher seroprevalence rate among male donors than the female blood donors might be due to risk behaviors of males, such as outside socialization, multiple sex relationships and may also be due to fewer females donating blood; hence fewer females are screened compared to males.

The prevalence of HCV in this study was 1.6% in blood donors. This study showed a similar prevalence rate with the study in Amhara and Tigray regional states, Ethiopia, 1.7% [15], in Dares salaam, 1.5% [13], in Abidjan Cote d'Ivoire, 1.5% [22], in Antananarivo, Madagascar, 1.6% [19] and in Nigeria, 1.5% [26]. However, this study showed higher prevalence than a study in Ethiopia, 0.7, 0.32 and 0.4% [16, 37, 39], in Netherland, 0.488% [10], Nepal, 0.64 and 0.48% [11, 29], in the University Clinics of Kinshasa of Democratic Republic of the Congo, 1.3% [9], Saudi Arabia, 0.4% [14], Istanbul, Turkey, 0.07% [28], in Delhi, 0.66% [31], in Kashan, Iran, 0.5% [32], in southeastern Anatolia, 0.64% [33], in India, 0.54 and 0.84% [34, 42]. But this HCV prevalence in this study lower than a study in Ethiopia, 13.3% [25], Cameroon, 4.8% [23], in Pakistan, 3.3, 5.14, 3.26, 2.61% [30, 35, 38, 40], in Nigeria, 6% [27], in Koudougou, 8.69% [36] and in Lahore, 7.69% [41]. This difference in prevalence of HCV from other studies might be due to the difference in characteristics of the study population, geographical distribution and diagnostic techniques.

The seroprevalence of HCV was detected in the age groups of 20–29, and 30–39 years was 0.7% (42/5983) & 0.55% (33/5983), respectively which is higher as compared to other age groups. The difference is statistically significant (*p* = 0.003). Which shows, the risk of HCV in the age group 20–29 increased by 93% compared with other age groups. This finding in line with other studies [20, 21]. The risk of HCV infection in young age increment might be due to a higher habit of alcohol drinking, having multiple sexual partners and intravenous drug users among young age groups.

HCV infection by gender distribution showed that male had a 1.5% prevalence which shows higher than their female counterparts, 0.15%. This finding similar to the finding of seroprevalence HCV among blood donors in Gondar [16] and in Madagascar [19]. High prevalence of HIV, HBV, and HCV co-infection was detected among blood donors and five cases showed the presence of three markers (HIV, HBV, and HCV). The HIV–HBV co-infection rate was 50%, the HIV–HCV co-infection rate was 24%, the HBV–HCV co-infection rate was 26% and HIV–HBV- HCV co-infection rate was 8.6%. It might be due to a similar mode of transmission, namely through sexual activity, blood and blood products, sharing of needles to inject drugs and co-infection with the impairment of the immune system.

The trends in transfusion-transmitted infections (TTIs) in blood donors tend to decrease in this study, HIV, HBV, and HCV went from 3.3, 5.2, and 1.4% in September 2014 – August 2015, 2.6, 5.2, and 2.3% in September 2015 – August 2016 and 1.7, 4.0 and 1.3% in September 2016 – September 2017 respectively, compared with those in the previous studies [13, 22]. But the seroprevalence of HCV was not constantly decreased in this study. The lower prevalence and decreasing trend TTIs in this study might be due to the increment of awareness on the prevention measures, the disease, and modes of prevention.

## Conclusion

A large percentage of the blood donors harbor transfusion-transmissible infections, 8.2% with at least one screened pathogen and 0.96% with multiple infections. The prevalence of TTI infection high for all blood donors especially for age groups such as 20–29 and 30–39 years. Therefore, strict selection of blood donors with the emphasis on getting voluntary donors and highly sensitive and specific tests for screening of donors’ blood for HIV, HBV, and HCV using standard methods are highly recommended to ensure the safety of blood for the recipient. The prevalence of HIV-HBV, HIV-HCV, and HBV-HCV co-infection need to be studied on a larger scale for a better understanding of the impact on clinical condition and treatment response.

## Abbreviations

AIDS: Acquired Immuno Deficient Syndrome
ART: Anti-retroviral Therapy
AZT: Azidothymidine
HAART: Highly Active Antiretroviral Drug
HBsAg: Hepatitis B Surface antigen
HBV: Hepatitis B virus
HCV: Hepatitis C Virus
HIV: Human immunodeficiency virus
MACS: Multi-center AIDS Cohort Study
RNA: Ribonucleic Acid
TTI: transfusion-transmissible infections
